# Supplementary figures and images for: Caecal microbiota compositions from 7-day-old chicks reared in high-performance and low-performance industrial farms and systematic culturomics to select strains with anti-Campylobacter activity
Source: PLoS One. 2020 Aug 24;15(8):e0237541. doi: 10.1371/journal.pone.0237541 (PMC7446796; doi:10.1371/journal.pone.0237541)

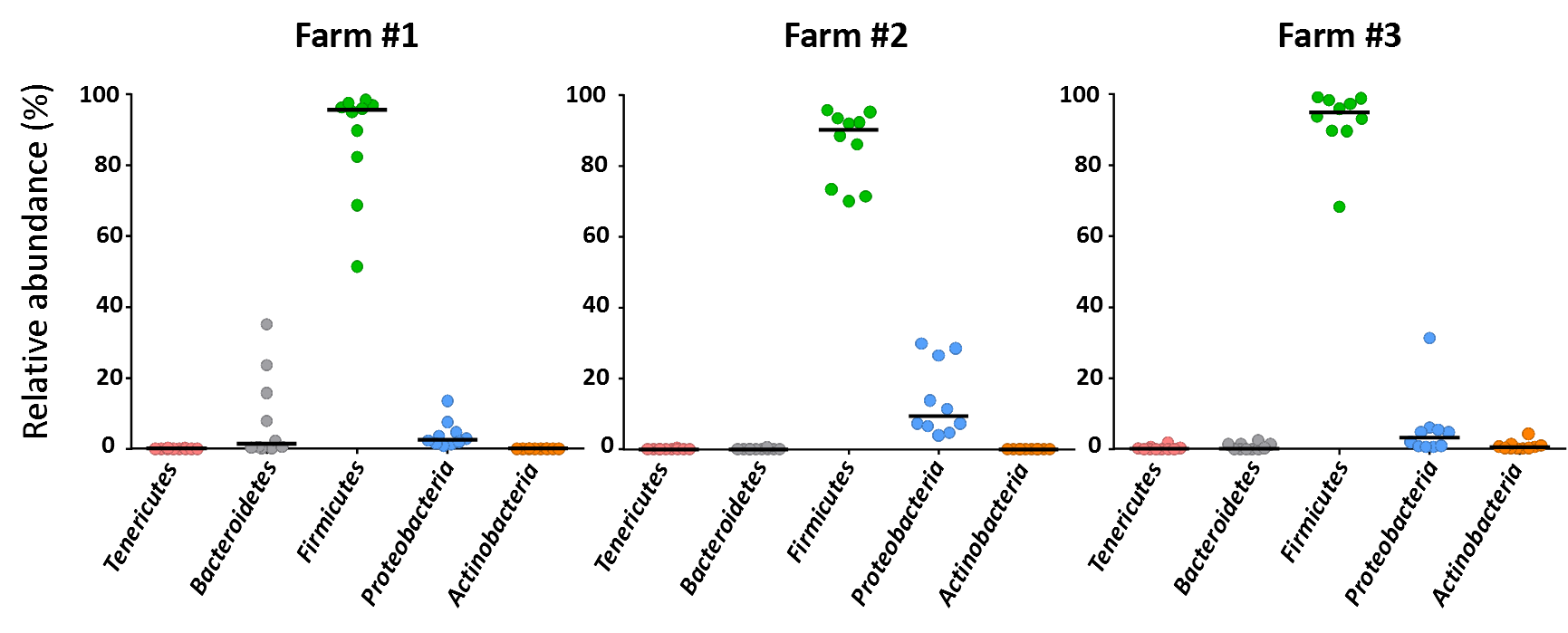

Supplement: S1 Fig — n = 10 points for each farm. (TIF) [file pone.0237541.s001.tif]

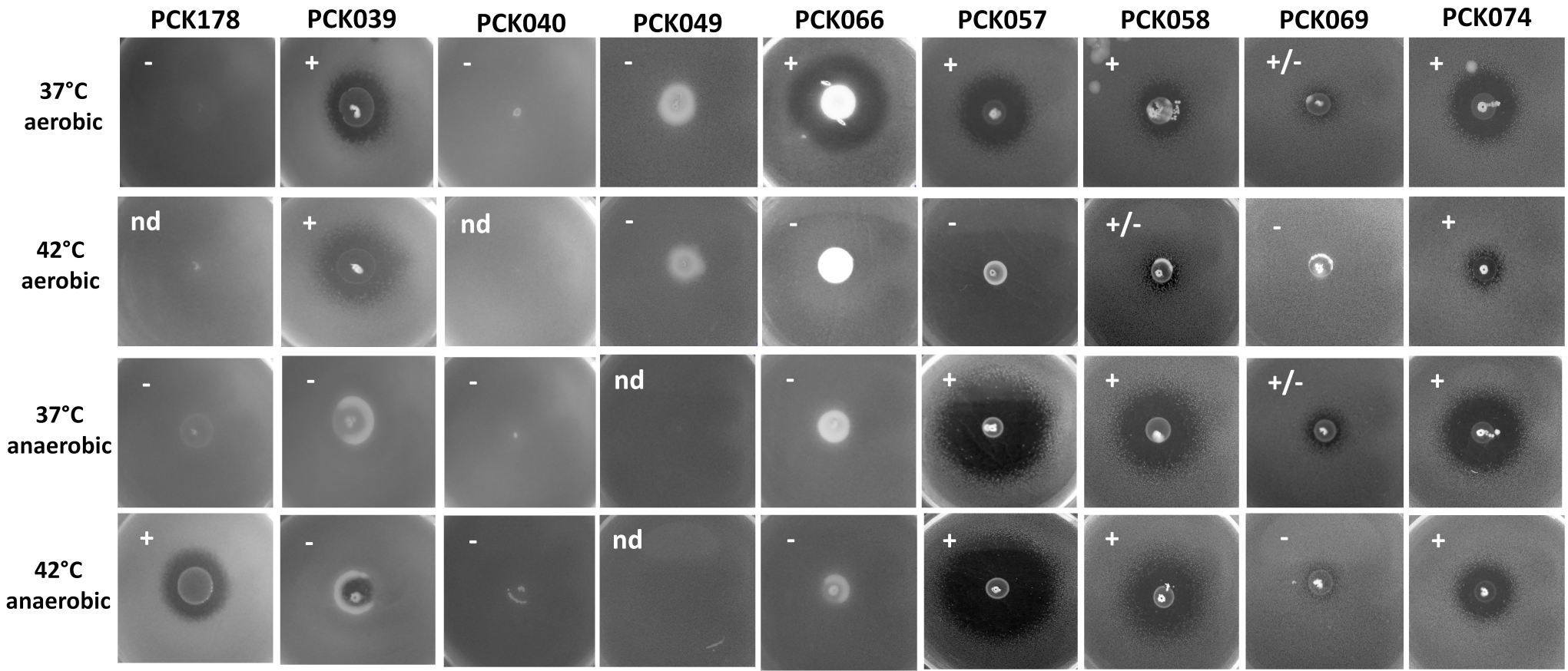

Supplement: S2 Fig — Inhibition diameters were measured and scored with the following parameters: (+) for inhibition diameters ≥ 2 mm, (+/-) for 0.1 to 1.9 mm, (-) when no inhibition was observed. nd: no done due to the absence of growth in tested conditions. (TIF) [file pone.0237541.s002.tif]

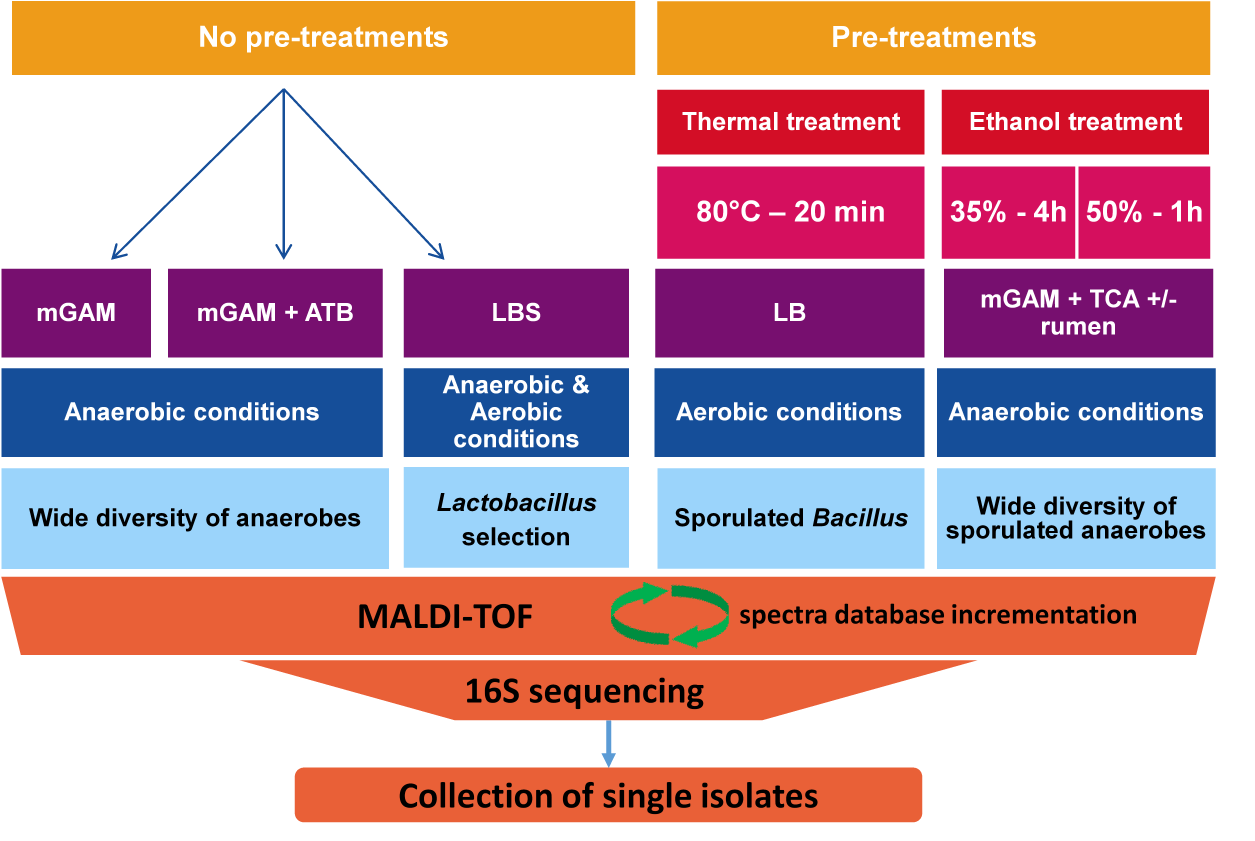

Supplement: S3 Fig — Caecal content was diluted in reduced PBS and then part of the suspension was directly seeded on mGAM with or without antibiotics and on LBS agar plates that were incubated anaerobically (mGAM) and/or aerobically (LBS) to allow recovery of a variety of anaerobes and of Lactobacillus species, respectively. Another part of the suspension was subjected to heat or to ethanol selection treatment and then seeded on LB or on mGAM complemented with sodium taurocholate and rumen fluid before incubation in aerobic or anaerobic conditions to allow recovery of sporulated Bacillus species or sporulated anaerobes, respectively. (TIF) [file pone.0237541.s003.tif]
